# “It doesn’t make any sense to even try”: the disruptive impact of COVID-19’s first wave on people with chronic pain using medical cannabis in New York

**DOI:** 10.1186/s42238-023-00180-1

**Published:** 2023-03-29

**Authors:** Yuval Zolotov, Jacinta Lomba, Megan Ghiroli, Mariya Masyukova, Julia H. Arnsten, Joanna L. Starrels, Jonathan Ross, Chinazo O. Cunningham, Deepika E. Slawek

**Affiliations:** 1 Division of General Internal Medicine, Department of Medicine, Montefiore Medical Center/Albert Einstein College of Medicine, 3300 Kossuth Ave, Bronx, NY 10467 USA; 2 Department of Family and Social Medicine, Montefiore Medical Center/Albert Einstein College of Medicine, Bronx, NY USA

**Keywords:** Medical cannabis, Medical marijuana, Chronic pain, COVID-19

## Abstract

**Background:**

The COVID-19 pandemic disrupted health care but it is unknown how it impacted the lives of people using medical cannabis for chronic pain.

**Objective:**

To understand the experiences of individuals from the Bronx, NY, who had chronic pain and were certified to use medical cannabis during the first wave of the COVID-19 pandemic.

**Methods:**

We conducted 1:1 semi-structured qualitative telephone interviews from March through May 2020 with a convenience sample of 14 individuals enrolled in a longitudinal cohort study. We purposively recruited participants with both frequent and infrequent patterns of cannabis use. Interviews addressed the impact of the COVID-19 pandemic on daily life, symptoms, medical cannabis purchase, and use. We conducted a thematic analysis, with a codebook approach, to identify and describe prominent themes.

**Results:**

Participants’ median age was 49 years, nine were female, four were Hispanic, four were non-Hispanic White, and four were non-Hispanic Black. We identified three themes: (1) disrupted access to health services, (2) disrupted access to medical cannabis due to the pandemic, and (3) mixed impact of chronic pain on social isolation and mental health. Due to increased barriers to health care in general and to medical cannabis specifically, participants reduced medical cannabis use, stopped use, or substituted medical cannabis with unregulated cannabis. Living with chronic pain both prepared participants for the pandemic and made the pandemic more difficult.

**Conclusion:**

The COVID-19 pandemic amplified pre-existing challenges and barriers to care, including to medical cannabis, among people with chronic pain. Understanding pandemic-era barriers may inform policies in ongoing and future public health emergencies.

**Supplementary Information:**

The online version contains supplementary material available at 10.1186/s42238-023-00180-1.

## Background

New York City (NYC) was the epicenter of the COVID-19 pandemic in the USA from March to May 2020. It bore the brunt of cases, hospitalizations, and deaths from COVID-19 in the USA during this first wave, and the Bronx was disproportionately impacted (Bialek et al. 2020). The Bronx is home to the poorest congressional district in the USA and is ranked among the worst in health outcomes in New York State (NY) (Rankings et al. 2022). Of the more than 15,000 people who died from COVID-19 in NYC from March to May 2020, 3,300 were in the Bronx (New York City Department of Health and Mental Hygiene 2022). Other boroughs in NYC had higher rates of COVID-19, but the Bronx had the most severe outcomes, including hospitalizations and deaths. Economic disparities were also exacerbated during the pandemic. For example, rates of unemployment in the Bronx were the highest in NYC in 2020 (Office of the New York State Comptroller 2021).

Chronic pain is highly prevalent in the USA and is considered a major public health crisis. It is a leading cause of reduced quality of life and healthcare spending (Gaskin and Richard 2012; Vos et al. 2015). For decades, chronic pain was commonly managed with long-term opioid therapy. With the worsening epidemic of opioid overdose, the use of long-term opioid therapy for chronic pain is now discouraged (Dowell et al. 2016). Many patients and providers have sought alternative therapies, including medical cannabis. NY’s medical cannabis program was implemented in 2016 and was enthusiastically adopted by patients and providers, particularly to manage pain. In the first 2 years of NY’s medical cannabis program, over 70% of patients certified for medical cannabis did so for chronic pain (New York City Department of Health and Mental Hygiene 2022). By February 2022, over 120,000 patients had been certified for medical cannabis use in NY (Office of Cannabis Management 2022).

Medical cannabis remains inaccessible for many patients because of financial and structural barriers (Ross et al. 2022). In NY as in other states, medical cannabis products are not covered by insurance and patients must pay for them with cash rather than credit or debit cards (New York City Department of Health and Mental Hygiene 2022). Certifying providers in NY are less likely to be located in areas with Black residents than in areas with other racial and ethnic compositions, and medical cannabis dispensaries are more highly concentrated in highly educated neighborhoods than in neighborhoods with less educated population (Cunningham et al. 2022).

The first wave of the COVID-19 pandemic suddenly and severely disrupted everyday life, including and especially healthcare and other services. Individuals in NYC were encouraged not to leave their homes or use public transit, outdoor recreational activities were discouraged, and any gatherings deemed “non-essential” were canceled by state decree (State et al. 2020). Outpatient medical services and clinics closed to reduce the risk of exposure and resources were shifted to treating severely ill, hospitalized patients with COVID-19 (Chudasama et al. 2020). Among outpatient services that continued, most in-person visits ceased and transitioned to telehealth (El-Tallawy et al. 2020). In NY, medical cannabis dispensaries were considered an essential service and remained open during the first wave of the COVID-19 pandemic (Levin 2020), with expanded delivery services and curbside pickups (Angell 2020). It is not known how these changes impacted patients, particularly with messages that encouraged them to stay at home and limit interactions with others. No studies have examined the perspectives of patients with chronic pain who use medical cannabis during the COVID-19 pandemic. To address this gap, we conducted a qualitative study to understand the experiences of individuals in NYC during the first wave of the COVID-19 pandemic who had chronic pain and were certified for medical cannabis.

## Methods

We conducted qualitative, semi-structured individual interviews with a subgroup of participants of the 18-month longitudinal cohort Medical Marijuana and Opioids (MEMO) Study (NCT03268551). This study is reported in accordance with the Consolidated criteria for Reporting Qualitative research (COREQ) Checklist (Tong et al. 2007).

MEMO participants (*n* = 243) were recruited from a primary care-based medical cannabis program at Montefiore Medical Center in the Bronx, NY, and four NYC-based medical cannabis dispensaries operated by Vireo Health and Columbia Care. Inclusion criteria for the MEMO Study were as follows: (1) ≥ 18 years old, (2) fluency in English, (3) new certification for medical cannabis in NY within 90 days, (4) no medical cannabis use in the 6 months prior to certification, (5) medical cannabis certification qualifying condition of “chronic or severe pain” or “pain that degrades health and functional capability as an alternative to opioid use,” and (6) use of prescribed or illicit opioids in the past 30 days. Exclusion criteria were as follows: (1) inability to provide informed consent, (2) inability to complete study visits over 18 months, (3) unique pain syndromes (i.e., cancer, multiple sclerosis), (4) terminal illness, and (5) current or prior psychotic disorder. For the current study, additional inclusion criteria were as follows: initiation of medical cannabis use at least 3 months prior to interview and enrollment in the MEMO Study for at least 3 months. To understand potential differences based on the frequency of medical cannabis use, we purposively sampled MEMO participants with varied use patterns: infrequent use (typically 0–3 days out of 14), occasional use (typically 5–10 days out of 14), and frequent use (typically 11–14 days out of 14). Detailed methods for the MEMO Study were published previously (Cunningham et al. 2020).

For this qualitative study, MEMO participants were called by a member of the research team (JL) by phone. She explained her role in the MEMO Study and in the MEMO Qualitative Study, described the purpose of the study, and asked if they would be willing to participate. At the time of the interviews, JL was a study coordinator with the MEMO Study with graduate-level training in qualitative interviewing. Some individuals recruited for the qualitative study interacted with JL during prior MEMO study activities. If they were interested, participants provided their informed consent via telephone and continued with the interview. All interviews were conducted by phone for the safety of the participant and the interviewer.

The interview guide was informed by the socioecological model (Bronfenbrenner 1989). Interviews focused on reasons for using medical cannabis, experiences with obtaining and using medical cannabis, the impact of medical cannabis use on their health, and issues impacting access to medical cannabis after certification, including cost. Examples of questions included “What was your experience obtaining your medical cannabis?,” “What does medical cannabis do for you?,” “What do others, including your medical providers, think about your medical cannabis use?,” and “How does medical cannabis affect your medical conditions?” The interview guide was refined iteratively. We included questions on how the pandemic impacted medical and psychiatric symptoms such as pain and anxiety, how participants coped with increased stressors of the pandemic, and whether participants changed medical cannabis or other medication use such as opioids in response to the COVID-19 pandemic. The interview guide is included as Supplementary Material 1. All interviews lasted approximately 45 to 60 min and were audio-recorded and professionally transcribed. Participants received $40 compensation for their time.

Additional sociodemographic and clinical variables were collected during MEMO study visits using Audio Computer-Assisted Self-Interview (ACASI) technology. Sociodemographic variables included age, gender, race, employment (yes/no), and years of education. Clinical variables included locations of pain (Keller et al. 2004); pain intensity and interference (Pain, Enjoyment of Life and General Activity Scale [PEG-3; range 0–10, moderate/severe ≥ 5]) (Krebs et al. 2009); insomnia symptoms (Insomnia Severity Index [ISI; range 0–28, clinical/severe insomnia ≥ 15]) (Morin et al. 2011); anxiety symptoms (General Anxiety Disorder-7 [GAD-7; range 0–21, moderate/severe ≥ 10]) (Spitzer et al. 2006); and depressive symptoms (Patient Health Questionnaire [PHQ-9; range 0–27, moderate/severe ≥ 10]) (Kroenke et al. 2001).

**Fig. 1:**
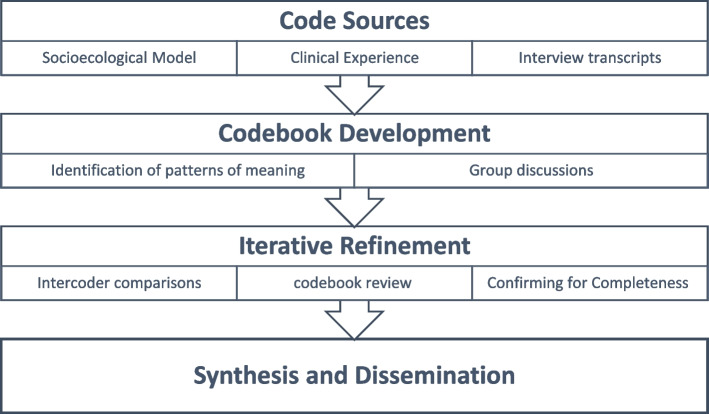
Analytic process

We analyzed the data iteratively using thematic analysis with a codebook approach (Braun and Clarke 2006; Braun and Clarke 2022). After being professionally transcribed, interview data were imported into Dedoose software (Hermosa Beach, CA) (Vaismoradi et al. 2013). We applied a hybrid of inductive and deductive approaches during the data analysis and the development of the codebook. The socioecological model guided the deductive component, while for the inductive part, we (DES, JL, MM, MG) reviewed the first three interviews to identify patterns of meaning across the interviews and generate a preliminary list of codes. With group consensus, we organized the list of codes into a preliminary coding scheme. Through regular discussions, codes were iteratively added, modified, merged, and eliminated based on discrepancies and constant comparison within and between codes. All transcripts were coded by two members of the research team using the final coding scheme and all disagreements were reconciled with the full coding team. Interviews were continued until no novel issues were identified and codes were meaningful enough so that additional data collection would be redundant (i.e., thematic saturation) (Kerr et al. 2010; Hennink et al. 2017). Figure 1 provides an illustration of the analytic process.

We analyzed quantitative data by conducting simple frequencies, means, and medians to describe sociodemographic and clinical variables.

## Results

Of 30 MEMO participants called, eight declined and 22 consented to participate in qualitative interviews. Of these, 14 interviews were conducted during the time frame of interest (the first wave of the COVID-19 pandemic) and are included in this analysis. The median age of participants was 49 years (interquartile range (IQR): 27–82). Nine participants were female, four were Hispanic, four were non-Hispanic White, four were non-Hispanic Black, and one identified as other race. Most participants were unemployed (*n* = 9) and had completed at least 12 years of school (*n* = 12). Five participants reported either prescribed or non-prescribed opioid use during the 30 days prior to their survey visit closest to the qualitative interview, and only 2 participants used tobacco. The most commonly reported pain locations were the back (*n* = 11) and the knee (*n* = 10); nine participants reported having pain in four or more body sites. The median PEG-3 score was 7.66 (IQR: 0–9.66), with 12 participants reporting moderate to severe pain. Five participants reported clinically significant insomnia, four participants reported moderate to severe symptoms of anxiety, and six participants reported moderate to severe symptoms of depression. Four participants reported infrequent medical cannabis use, 5 reported occasional medical cannabis use, and 5 reported frequent medical cannabis use. Overall, the demographic and clinical characteristics of participants interviewed during the COVID-19 first wave (*n* = 14) and participants who declined or were interviewed prior to the COVID-19 pandemic (*n* = 16) were similar (see Table 1).

**Table 1:** Sample characteristics

	Participants *N* = 14	Nonparticipants *N* = 16
Age, median (IQR)	48.5 (27–82)	53 (26–81)
Gender, n (%)		
Male	4 (30)	5 (31.3)
Female	9 (64)	11 (68.8)
Transgender	1 (7)	0 (0)
Race, *n* (%)		
Hispanic	4 (28.5)	3 (18.7)
Non-Hispanic White	4 (28.5)	10 (62.5)
Non-Hispanic Black	4 (28.5)	3 (18.7)
Non-Hispanic Other	1 (7.1)	0 (0)
Prefer not to answer	1 (7.1)	0 (0)
Employed, *n* (%)		
Yes	5 (36)	6 (37.5)
No	9 (64)	10 (62.5)
Education, *n* (%)		
< 12 years	2 (14)	3 (18.8)
≥ 12 years	12 (86)	13 (81.3)
Tobacco smoking, *n* (%)		
Yes	2 (14)	4 (25)
No	12 (86)	12 (75)
Using opioids, *n* (%)		
Yes	5 (36)	10 (62.5)
No	9 (64)	6 (37.5)
Medical cannabis use frequency, *n* (%)*		
Infrequent use	4 (28)	2 (12.5)
Occasional use	5 (36)	6 (37.5)
Frequent use	5 (36)	8 (50)
Pain location		
≥ 4 body sites	9 (64)	8 (50)
Head/neck	7 (50)	9 (56.3)
Arm/elbow/hand	6 (43)	8 (50)
Shoulder	7 (50)	7 (43.8)
Back	11 (79)	13 (81.3)
Knee	10 (71)	7 (43.8)
Leg	8 (57)	8 (50)
Pain intensity and interference (PEG-3), median (IQR)	7.66 (0–9.66)	
Insomnia^a^, *n* (%)		
No clinically significant insomnia	5 (36)	5 (31.3)
Subthreshold insomnia	4 (29)	(37.5)
Clinical insomnia (moderate severity)	3 (21)	3 (18.8)
Clinical insomnia (severe)	2 (14)	2 (12.5)
Anxiety^b^, *n* (%)		
Minimal anxiety	7 (50)	7 (43.8)
Mild anxiety	3 (21)	3 (18.8)
Moderate anxiety	0 (0)	4 (25)
Severe anxiety	4 (29)	2 (12.5)
Depression^c^, *n* (%)		
Minimal depression	5 (36)	3 (18.8)
Mild depression	3 (21)	5 (31.3)
Moderate depression	2 (14)	3 (18.8)
Moderately severe depression	4 (29)	4 (25)
Severe depression	0 (0)	1 (6.3)

*IQR* interquartile range

^a^Insomnia Severity Index

^b^GAD-7 Scale

^c^PHQ9 Scale

^*^Infrequent use = 0–3 days out of 14 on most web-based surveys, occasional use = 4–10 days out of 14 on most web-based surveys, frequent use = 11–14 days out of 14 on most web-based surveys

We identified three themes: (1) disrupted access to health care, (2) disrupted access to medical cannabis, and (3) the mixed impact of chronic pain on social isolation and mental health. In addition to the quotes presented in the text below, other quotes that support our findings are included in Supplementary Material 2.

### Theme 1: Disrupted access to healthcare

Participants described disruption of the continuity of care and overall access to healthcare services due to the COVID-19 pandemic. They were often unable to reach their usual clinicians or receive timely care for their symptoms, including pain as well as other acute, sometimes life-threatening, conditions. Public health messaging during the first wave stressed the importance of staying home unless experiencing symptoms needing emergent evaluation. Patients were therefore often tasked with determining for themselves whether they should seek out urgent or emergent care without input from their primary care providers. This proved particularly challenging for participants in this study who experienced chronic pain, were living with multiple medical and psychiatric comorbidities, and often faced already-existing socioeconomic and race/ethnicity-related barriers to care. For example, one participant recounted:

I started having increased shortness of breath and an increased heart rate. And my oxygen levels have gone down a little bit the night before. So, I think I probably have pneumonia and I need to be seen. And I emailed the doctor and, you know, by the time they answered me, I had already finished with urgent care. (Participant #4)

During the first wave of the pandemic, routine in-person care and procedures determined to be “elective” were canceled or indefinitely postponed by state decree. Participants described how their symptoms worsened as a result of inability to access care. For example, one participant said:

I’m very much in pain now, I can hardly walk. That’s why I went to the pain management doctor last week, and he said that there’s a restriction on surgical procedures in the state right now because of the coronavirus, that he can’t – he couldn’t help me. He told me to see him again in a couple of months. (Participant #5)

To reduce the risk of exposure and spread of COVID-19, many healthcare services transitioned from in-person visits to telemedicine. While this allowed patients to interact with their clinicians without potentially risky face-to-face contact, some participants struggled to access adequate and timely care and described limited communication with healthcare providers. Some participants lacked digital literacy, making telemedicine impractical and unobtainable for them and adding more barriers to care and subsequent frustration. Some even felt that they were ostracized and unwanted in the setting where they usually received medical care.

No one’s answering back. It’s just – it’s frustrating, you know… Everyone is either working from home or being displaced or, you know, the doctors aren’t even at the doctor’s office. And I’m trapped in a room with no fax machine and no scanner, and I have to teach myself how to do these things with my phone. And I have limited mental capacity and it’s just – it’s frustrating. It’s very frustrating. (Participant #4)

### Theme 2: Disrupted access to medical cannabis

Participants described multiple barriers to access to medical cannabis, including many that predated the COVID-19 pandemic, for example transportation and time barriers related to accessing the few existing dispensaries. In particular, many participants identified cost as a major impediment to medical cannabis access, especially given the lack of insurance coverage for medical cannabis products.

The cost is a lot. It’s expensive…. So, yeah, if you can’t afford it and your insurance is not paying for it… like, my daughter for instance, my daughter is really sick, she has neuropathy in all of her body. She got a [medical cannabis] card, took her with the card, but she’s on SSI, she can’t afford to go. She can’t afford to go get the marijuana because she can’t afford to pay for it. (Participant #13)

These barriers grew more acute during the pandemic, including the limited availability of medical cannabis dispensaries across geographic locations and cost barriers to purchasing medical cannabis. Although dispensaries were deemed an essential service in NY and were therefore allowed to remain open for business, most were open only for pick-up or offered deliveries at additional costs to encourage social distancing. Participants had difficulty navigating these barriers and expressed difficulties accessing their medical cannabis.

[In the past,] I’d managed to get a friend to drive me to a dispensary to get a refill, and thank goodness. Now, everyone, I mean, some places are like, “We can deliver.” But I can’t actually afford that delivery. It’s too expensive, you know? Other places are like, “We’re only open for pickup.” And I’m like, “Well, I can’t go out, pick it up, because I can’t walk.” … Now, my friends can’t come out and drive me anymore, so, I can’t go anywhere. (Participant #14)

In response to increased barriers to medical cannabis during the pandemic, participants responded by reducing or even stopping medical cannabis use, or switching to unregulated cannabis. Participants who experienced financial hardship related to the pandemic reported reducing medical cannabis use or stretching out their supply to reduce cost and make ends meet. One participant stated:

Since this coronavirus, money’s been, you know, on the low. I still have [cannabis] oil. So, I’ve been kind of like limiting the oil a little bit and I only take it when I feel like I can’t endure the pain. So, I take it -- rather than just take it whenever, you know, actual like, you know, -- you have to take it like how you take your pills. (Participant #10)

Another way in which participants responded to increased barriers to medical cannabis during the pandemic was supplementing their medical cannabis with, or switching entirely to, unregulated cannabis.

The delivery fees are very expensive. So, that kind of puts me in a position to where, I know, I kind of buy it off the books, so to speak, and get marijuana instead of medical marijuana. (Participant #12)

Several participants noted that they switched to medical cannabis from unregulated cannabis to use cannabis products that were safer and that were more socially acceptable. Medical cannabis products in NY were oil-based during these interviews and were described as causing less of an odor and provoking less stigma from community members.

As far as just the coughing…, I’m going to stay away from doing that, because .. people are automatically assuming that you have coronavirus, when it’s really smoking. But … with the vape, it’s not a lot of coughing. (Participant #12)

Switching back to unregulated cannabis was a source of disappointment and increased risk of becoming exposed to cannabis products contaminated with heavy metals, pesticides, or other drugs and cannabinoid content that was either mislabeled or not labeled at all.

### Theme 3: Mixed impact of chronic pain on social isolation and mental health

Participants’ refractory chronic pain and comorbidities shaped the way they experienced the COVID-19 pandemic’s first wave. Participants described that their illness and impaired mobility had resulted in social isolation well before the pandemic. In some ways, they felt equipped to cope with the social isolation imposed by the pandemic.

I’m at home anyway. I’ve been social distanced for two years now. You know? I mean, I don’t go out. I don’t go anywhere. I have a home health aide and I just don’t get out and neither does my wife anymore. (Participant #1)

Some participants described how learning to live with their chronic health conditions and the resultant social isolation contributed to a sense of resilience, so much so that participants felt less impacted by the loneliness experienced by many during the pandemic. Participants expressed hope that the social isolation faced by the general population during the first wave of COVID-19 would allow others to understand the daily experience of chronic pain patients:

I’m welcoming the world to my life. The whole quarantine and social distancing thing – it was like, this is my life anyway. (Participant #11)

In contrast, some participants described worsened mental health symptoms during the pandemic, ranging from boredom and agitation to exacerbation of depression and anxiety symptoms. They related this to social isolation and the uncertainties of the pandemic:

I can say the anxiety and depression, yeah… It’s gotten worse. I definitely can say that… You know, you can’t get around, you can’t see people, you can’t talk, you can’t release. So you just holding in everything. (Participant #9)

Participants who lived alone were especially vulnerable to anxiety that was intertwined with loneliness:

I’m by myself, like, 95% of the time. So, that, in itself, causes anxiety. (Participant #3)

Many participants described their fear of becoming infected with COVID-19. Of the 14 interviewed, three participants were either sick with COVID or recovering from it during the interview. These participants were attuned to the health risks of COVID and grateful that they did not require admission to the hospital, but they also felt guilt that they were not on the frontlines with their communities and co-workers who were facing the pandemic.

I see my coworkers suffering and I can’t be there. I have this guilt that I’m here in a bed and they’re working and doing the best they can and I’m not there. (Participant #4)

Those with medical comorbidities worried that they would be particularly vulnerable to severe manifestations of the virus; others described concern about how they would be able to maintain their daily responsibilities if they became sick.

When I do go outside, I wear the mask around my face, because with the autoimmunity disease I have a really low immune system… Of course I get scared. But I think about myself and try to avoid getting sick and then, being in the hospital with all the sick people and then getting exposed. So, I do worry much about that. (Participant #10)

More generally, participants pointed to the terrible circumstances caused by the pandemic. Several expressed their sorrow for so many who got sick and for many others who died, as well as for the societal and economic situation. Living in NYC, which was an early epicenter of the pandemic, participants were likely to know people who got sick with, or who died of, COVID-19. One participant said:

What’s really bothering me is the amount of people who are dying… So many people I know whose relatives or somebody has died because of this; and my godson, he’s 39, he’s been on a ventilator for a week now. (Participant #2)

## Discussion

We conducted one of few qualitative studies to examine how the first wave of the COVID-19 pandemic impacted people with chronic pain, and this is the first study, to our knowledge, conducted among individuals who use medical cannabis for pain. The findings illustrate the unique experiences of people with severe and chronic pain who were certified for medical cannabis in NYC during the first wave of the COVID-19 pandemic. Despite medical needs resulting from their multiple medical and psychiatric comorbidities, participants experienced challenges in accessing health care, including medical cannabis. Though participants considered medical cannabis an important aspect of their pain management, the challenges in obtaining it before the COVID-19 pandemic were exacerbated by its onset. In addition, while participants felt well-acquainted with social isolation due to chronic pain and other comorbidities, many experienced worsening mental health symptoms during the pandemic. Our findings provide a nuanced understanding of the experiences of a patient population struggling to access relief during a historic public health crisis.

Our findings demonstrate how the COVID-19 pandemic intensified pre-existing challenges among people with chronic pain. For example, participants were isolated because of their medical conditions regardless of the pandemic—and this was amplified due to the pandemic. These findings are similar to those from a qualitative study of people with chronic pain from Canada (Dassieu et al. 2021), reinforcing the conclusion that a vulnerable patient population became even more susceptible to deteriorating conditions because of COVID-19. They are also consistent with analyses that found that mental health outcomes worsened during the COVID-19 pandemic among other populations, such as pregnant and breastfeeding women and adults in general (Ceulemans et al. 2021; Jacob et al. 2021).

Recent quantitative studies showed a mix of increased, decreased, and unchanged use of medical cannabis during the pandemic (Wang et al. 2021; Vidot et al. 2020; Boehnke et al. 2021). Our qualitative findings shed light on potential reasons for such altered patterns of use. Along with logistical barriers to accessing medical cannabis imposed by social distancing measures to mitigate the pandemic, cost was a prominent concern that worsened access to medical cannabis. While previous studies pointed to cost as a significant challenge to certified patients prior to the pandemic (Zeng et al. 2021; Zhu et al. 2021), this burden was worsened due to newly added delivery costs and to financial vulnerability that participants experienced during the pandemic. In addition, the pandemic exacerbated disparities in access to medical cannabis services which previous studies identified in NY before the pandemic (Cunningham et al. 2022).

Overall, participants described three main strategies for coping with increased barriers to medical cannabis: reducing their purchase and consumption, eliminating use altogether, or switching to unregulated cannabis. For individuals with severe and refractory pain, such as our sample, these may result in worsened pain or associated outcomes. Future studies may attempt to examine this association. Furthermore, substitution of medical cannabis with unregulated cannabis could reduce safety and effectiveness for symptom relief because it lacks quality control.

This study has several limitations. The interviews took place in the Bronx, NYC, during the first wave of the COVID-19 pandemic. While participants’ responses may have been related to whether they (or others in their household) were infected with COVID-19, the availability of COVID-19 tests at that time was extremely limited, and we were unable to obtain valid data about their infection status. Given the considerable variability in COVID-19 epidemiology and impact across locations and communities, our findings may not be generalizable to other settings or points in time. Participants were recruited from a longitudinal cohort study that examines the opioid-sparing potential of medical cannabis, which could also limit generalizability. During the qualitative interviews, participants did not mention switching to other pain medications due to the lack of availability of medical cannabis. Interviews were conducted by a member of the MEMO Study team. While this provided many participants comfort and ease of discussion because they knew the interviewer previously, it may have led to desirability bias during the interviews.

This study contributes to the limited literature on experiences of chronic pain patients who use medical cannabis. In the same way that the COVID-19 pandemic has highlighted longstanding societal disparities in healthcare and other arenas, our findings describe how the pandemic worsened barriers to care for patients with severe and chronic pain who may have high disease burdens and are prone to negative health outcomes (Fine 2011). While pre-pandemic qualitative studies described how patients perceived cannabis as beneficial in managing their pain, they did not capture the many barriers to accessing cannabis in a rapidly changing regulatory atmosphere, aside from stigma and legality (Bottorff et al. 2013; Page and Verhoef 2006; Coomber et al. 2003; Lavie-Ajayi 2019). Other studies, which focused on barriers to access, did not use qualitative methods and thus failed to capture a nuanced understanding of patients’ perspectives (Cooke et al. 2019; Singh et al. 2019; Piper et al. 2017; Capler et al. 2017).

Given that COVID-19 continues to be a health threat and the potential for more health crises (e.g., mPox), our findings have policy and clinical implications and may guide future studies. Deeming medical cannabis an essential service may not be enough to ensure access to this service, and policymakers should consider system-level changes to improve access to this care. For example, dispensaries should be located across more diverse areas and policies should be made to reduce the price of medical cannabis; NY is attempting to do so through taxation of nonmedical cannabis. Providers should be aware that certifying patients, by itself, is necessary but not sufficient and that access to medical cannabis may be hindered by many systemic barriers. Establishing outreach programs for vulnerable patients with chronic pain could potentially protect them from worsening health outcomes in times of crisis. Tailored interventions may be used to target social isolation and associated mental health conditions among patients with chronic pain who are at risk. Equitable access to medical cannabis is needed to avoid exposure of patients to unregulated products and to prevent their potential interaction with the justice system. Future research on patients with chronic pain certified for medical cannabis is encouraged to better understand the role that cannabis may have as part of their medical regimens, and also to shed light on the factors that limit access to this treatment.

In conclusion, this qualitative study revealed numerous ways in which the COVID-19 pandemic impacted individuals with chronic pain who are certified for medical cannabis in NY. The COVID-19 pandemic, as well as mitigating measures, intensified pre-existing disparities. Access to healthcare services, specifically to medical cannabis, became more challenging. Limited access to medical cannabis resulted in altered patterns of use and in some cases led to use of unregulated cannabis. Understanding how patients were impacted in the very beginning of the pandemic may contribute to preparing for, and coping with, future public health emergencies.

## Supplementary Information

**Additional file 1.** MEMO Qualitative Interview Guide.

**Additional file 2.** Additional quotes.

## Data Availability

The datasets generated and/or analyzed during the current study are not publicly available due to patients’ privacy.
