# Supplementary material for: “It doesn’t make any sense to even try”: the disruptive impact of COVID-19’s first wave on people with chronic pain using medical cannabis in New York
Source: J Cannabis Res. 2023 Mar 29;5:10. doi: 10.1186/s42238-023-00180-1 (PMC10049907; doi:10.1186/s42238-023-00180-1)
Supplement: Supplementary file 1 — Additional file 1. MEMO Qualitative Interview Guide. [file 42238_2023_180_MOESM1_ESM.docx]

**Supplementary Material 1: MEMO Qualitative Interview Guide**

**Introduction**

Thank you for agreeing to participate in today’s interview. To briefly describe the purpose of today’s interview, we are interested in understanding what your experience has been with medical cannabis and other medications that you take for the same symptoms. If there are any questions that I ask during our discussion that you are uncomfortable answering you can choose to skip the question. Our discussion will remain confidential. To maintain confidentiality, please try to refrain from using your name while we are recording. If you do happen to use your name, it will be redacted in transcripts.

1. Tell me about why you decided to use medical cannabis to treat your symptoms.
   1. What has your experience been like?
      1. [If participant does not bring this up on their own] What difficulties did you have in getting medical cannabis?
         1. Probe on price/cost of medical cannabis—Is cost ever difficult for you? How does the cost of medical cannabis differ from your other medications? Have you come across any coupons or discounts? Could you tell me about them? How did this affect what you bought?
      2. What things made it possible for you to get your medical cannabis? (e.g. How did you find your doctor? What was the process of getting your certification like? Walk me through the process of finding your doctor all the way to getting your medical cannabis at the dispensary.)
   2. What does medical cannabis do for you?
      1. How does it affect your symptoms?
      2. Have you experienced any negative effects from medical cannabis?
      3. Have you used “street” cannabis or recreational cannabis prior to using medical cannabis? How did that experience differ from your experience with medical cannabis [be sure to probe on this—get specific details on the differences between street and medical; intention, effect on symptoms]?
         1. Are you still using recreational cannabis? Why? [how is it different than medical? When do you decide to use recreational versus medical?]
   3. How are you using your medical cannabis?
      1. How did you get to this medical cannabis routine?
      2. Have you found some products or formulations to be more or less helpful for you? How so?
   4. How do others in your life feel about your medical cannabis use? What about your medical providers?
      1. Do you think you are treated differently because of your cannabis use?
      2. Does this impact whether or not you take medical cannabis? Why? How?
      3. How does your primary care provider feel about your medical cannabis use? What makes you think that?
         1. [If HIV positive participant and not brought up earlier] Is your primary care provider also your HIV provider? If not, how do they feel about your medical cannabis use?
   5. How does your medical cannabis use affect your other medical conditions? How so?
      1. [If participant bring up having any of the following] Tell me more about how medical cannabis affects your PTSD (insomnia, anxiety, depression).
2. Since you started using medical cannabis, do you think it changed the way that you have managed your conditions or symptoms (e.g. pain, sleep, anxiety)? If so, why?
   1. Thinking back to before you started using medical cannabis (e.g., 1 year ago), what symptoms and conditions did you have that were most bothersome to your day-to-day life? What did you do to manage these symptoms?
   2. Now, thinking about the past 1-2 days, what symptoms do you have that are most bothersome to your day-to-day life? What do you do to manage these symptoms?
   3. Has there been a change? Why? How does medical cannabis fit into that picture?
3. Now I’m going to ask you a few questions that are specific to your experience since coronavirus or COVID-19 has become a global pandemic.

During this stressful time, many people have experienced increased anxiety, depression, and different people deal with this in different ways. For example—alcohol sales have increased during the COVID-19 pandemic.

- 1. Have any of your symptoms of anxiety (pain, PTSD, insomnia, depression) changed because of the COVID-19 pandemic? How so?
     1. For example, has it affected your sleep?
     2. Have any of your family or friends been infected? How does that affect how you feel?
     3. What about staying at home? Has that affected your symptoms? Have your habits changed? Your job? Medical appointments? Pharmacy visits?
  2. [if yes] Are you using medical cannabis to address this change in symptoms?
     1. How has your use changed?
     2. Has it affected your purchasing patterns?
     3. Are you using other methods of managing these symptoms?
  3. Has your medical care changed during COVID 19?
     1. Have you had telemedicine visits? How do you feel about these compared to your other visits? Do you feel it has changed how your medical problems are being managed?
  4. Does the coronavirus/COVID-19 pandemic make you feel differently about your medical cannabis use?
