# Supplementary material for: “It doesn’t make any sense to even try”: the disruptive impact of COVID-19’s first wave on people with chronic pain using medical cannabis in New York
Source: J Cannabis Res. 2023 Mar 29;5:10. doi: 10.1186/s42238-023-00180-1 (PMC10049907; doi:10.1186/s42238-023-00180-1)
Supplement: Supplementary file 2 — Additional file 2. Additional quotes. [file 42238_2023_180_MOESM2_ESM.docx]

| Supplementary Material 2. Additional quotes | | |
| --- | --- | --- |
| Theme | | Citations |
| System-level | Disrupted access to health care | *There's nothing that he can do at this point in time because of the virus, he's not allowed to perform surgical procedures, so he said, "Your best solution is to take medical marijuana until the virus is over, then you can come and see me and I can do a procedure on your back." (Participant #5)* |
|  |  | *Went there [to the doctor] expecting him to give me shots in my back, and he says, "I can't. I don’t even have a mask to put on. We're suspending all surgical procedures." They consider that a surgical procedure. So, I couldn’t even get my shots the other day. (Participant #5)* |
|  | Disruptive access to medical cannabis | *…The only way you can get it is to call and make an appointment to go… So I can’t get out. So it doesn’t make any sense to even try because I can’t get out.* *(Participant #2)* |
|  |  | *I won't do it until the beginning of next month, because I am in a situation right now where I live – until my next paycheck comes in for my retirement, it won't come in until April 1st, so I don’t have a – I don’t have the funding to go right now to get it.* *(Participant #5)* |
|  |  | *…I used the cannabis oil for now, I don’t have any more and I can't get any more because, you know, of what's going on.* *(Participant #2)* |
| Individual-level | Mixed impact of chronic pain on social isolation and mental health | *But that doesn’t really cause me too much anxiety, because I've been sick for so long, it's kind of a – it's almost like I’m been immunized about getting knocked over these things now, because I'm always sick. I've always been sick.* *(Participant #3)* |
|  |  | *Well, it’s tough because the whole thing with COVID is, you know, “Don’t go out.” And my whole thing before COVID was, “I can’t go out.” So, I would say COVID may have affected me the very least of anyone except that it’s – it feels like I’ve been in this quarantine for two months longer than everyone else.* *(Participant #14)* |
|  |  | *it's scary, it's very, very scary, and I have my mom here, and I have to protect her.* *(Participant #13)* |
|  |  | *I need my medicine, I've been without medicine this month. I need my medicine, like, I'm really, really anxious, and, like, when I go outside, I am freaking out, people are, like, disrupting my freaking personal space.* *(Participant #11)* |
|  |  | *The only thing that bothers me, is the fact that I am so tired of having to say ‘My condolences.’ … Dealing with the death…made me so anxious that I had to take one of my pills to relax and it made me sleep.* *(Participant #2)* |
|  |  | *Being that I have social anxiety, for some reason, even now, it's like, I’m getting anxiety over not being able to socialize. It's like, being told not to do it is freaking me out. It's almost like I enjoy being stuck at home, and now that it's not my choice, like, I can't choose to do it, it's overwhelming. It's very overwhelming. And it's so weird, because it's like, all my life, this is what I've done, and so, why is it a problem?* *(Participant #11)* |
